# Interleukin-2 and histamine in combination inhibit tumour growth and angiogenesis in malignant glioma

**DOI:** 10.1054/bjoc.2000.1354

**Published:** 2000-08-17

**Authors:** M Johansson, R Henriksson, A T Bergenheim, L-O D Koskinen

**Affiliations:** Departments of Oncology, Neurosurgery, Umeå University, Umeå, SE-901 85, Sweden

**Keywords:** IL-2, histamine, glioma, rat, microvascular density, angiogenesis

## Abstract

Biotherapy including interleukin-2 (IL-2) treatment seems to be more effective outside the central nervous system when compared to the effects obtained when the same tumour is located intracerebrally. Recently published studies suggest that reduced activity of NK cells in tumour tissue can be increased by histamine. The present study was designed to determine whether IL-2 and histamine, alone or in combination, can induce anti-tumour effects in an orthotopic rat glioma model. One group of rats was treated with histamine alone (4 mg kg^–1^s.c. as daily injections from day 6 after intracranial tumour implantation), another group with IL-2 alone as a continuous subcutaneous infusion and a third group with both histamine and IL-2. The animals were sacrificed at day 24 after tumour implantation. IL-2 and histamine in combination significantly reduced tumour growth. The microvessel density was significantly reduced, an effect mainly affecting the small vessels. No obvious alteration in the pattern of VEGF mRNA expression was evident and no significant changes in apoptosis were observed. Neither IL-2 nor histamine alone caused any detectable effects on tumour growth. Histamine caused an early and pronounced decline in tumour blood flow compared to normal brain. The results indicate that the novel combination of IL-2 and histamine can be of value in reducing intracerebral tumour growth and, thus, it might be of interest to re-evaluate the therapeutic potential of biotherapy in malignant glioma. © 2000 Cancer Research Campaign


*British Journal of Cancer* (2000) **83**(6), 826–832
© 2000 Cancer Research Campaign
doi: 10.1054/ bjoc.2000.1354, available online at http://www.idealibrary.com on IDEAL®

### Interleukin-2 and histamine in combination inhibit tumour growth and angiogenesis in malignant glioma

**M Johansson[1], R Henriksson[1], AT Bergenheim[2] and L-OD Koskinen[2]**

Departments of [1]Oncology and [2]Neurosurgery, Umeå University SE-901 85 Umeå, Sweden

**Summary** Biotherapy including interleukin-2 (IL-2) treatment seems to be more effective outside the central nervous system when compared to the effects obtained when the same tumour is located intracerebrally. Recently published studies suggest that reduced activity of NK cells in tumour tissue can be increased by histamine. The present study was designed to determine whether IL-2 and histamine, alone or in combination, can induce anti-tumour effects in an orthotopic rat glioma model. One group of rats was treated with histamine alone (4 mg $kg^{-1}$ s.c. as daily injections from day 6 after intracranial tumour implantation), another group with IL-2 alone as a continuous subcutaneous infusion and a third group with both histamine and IL-2. The animals were sacrificed at day 24 after tumour implantation. IL-2 and histamine in combination significantly reduced tumour growth. The microvessel density was significantly reduced, an effect mainly affecting the small vessels. No obvious alteration in the pattern of VEGF mRNA expression was evident and no significant changes in apoptosis were observed. Neither IL-2 nor histamine alone caused any detectable effects on tumour growth. Histamine caused an early and pronounced decline in tumour blood flow compared to normal brain. The results indicate that the novel combination of IL-2 and histamine can be of value in reducing intracerebral tumour growth and, thus, it might be of interest to re-evaluate the therapeutic potential of biotherapy in malignant glioma. © 2000 Cancer Research Campaign

**Keywords**: IL-2; histamine; glioma; rat; microvascular density; angiogenesis

Patients with malignant astrocytoma, the most common primary brain tumour, have a poor prognosis. Radical surgery is usually not possible due to the infiltrative growth of the tumour, and if combined with radiotherapy a median survival time of 10–12 months can be achieved. Despite many efforts using a pharmacotherapeutic approach, no major advantages have so far been seen. Some interesting, though controversial, results have been obtained with immunotherapy including interleukin-2 (IL-2) in various experimental systems, as well as in the clinical setting, for malignant glioma (Takai et al, 1988; Barba et al, 1989a; Weber et al, 1989; Lillehei et al, 1991; Fleshner et al, 1992; Saris et al, 1992; Danaila et al, 1993; Hayes et al, 1995; Glick et al, 1997; Kruse et al, 1997; Fathallah Shaykh et al, 1998).

The central role of the cytokine IL-2 in activating NK cells and tumour infiltrating lymphocytes (Caligiuri et al, 1993) has encouraged trials of IL-2 in patients with solid cancers such as malignant melanoma and renal cell carcinoma. The impact on overall survival has, however, been questioned (Ljungberg and Henriksson, 1997; Henriksson et al, 1998). The detailed mechanisms, which explain the poor outcome, have not been fully delineated. Patients with cancer are presumed to respond less to immune stimuli than healthy controls and several hypotheses have been proposed to explain the poor immunogenicity of tumours. Recent in vitro studies suggest that NK cells are only weakly activated by IL-2 in the presence of phagocytes, presumably due to the production of an inhibitory signal from phagocytes (Hellstrand et al, 1994*a*; 1994*b*). It has been shown that histamine abrogates the phagocyte-derived suppressive signal and thus facilitates the IL-2-induced NK cell-mediated killing of tumour cells (Hellstrand and Hermodsson, 1990; Hellstrand et al, 1994*a*; Asea et al, 1996). Interestingly, NK cell targets seem to be of special importance in cytotoxic immune response in rat glioma (Holladay et al, 1992). Moreover, it has also been demonstrated that histamine enhances the anti-tumour effects of IL-2 and irradiation in a rat prostatic carcinoma (Johansson et al, 1998).

In consideration of the above we found it of interest to investigate whether treatment with histamine in combination with IL-2 affects the growth of intracerebral malignant glioma in rats. In addition, since it is known that histamine and IL-2 both affect the vascular function by increasing permeability (Alexander et al, 1989; Nomura et al, 1994) we evaluated the possible effects on tumour microvessel function and morphology.

*Received 6 January 1999*
*Revised 8 May 2000*
*Accepted 11 May 2000*

*Correspondence to*: M Johansson

### MATERIALS AND METHODS

### Tumour model and animal care

The previously described intracerebral syngenic BT4C rat glioma model was used for the experiments (Bergenheim et al, 1994; Johansson et al, 1997). Briefly, BT4C rat glioma cells were suspended to a concentration of 20 000 cells per 5 μl in minimal essential medium (MEM, Flow laboratories, Scotland). Inbred BDIX rats were anaesthetized with a 1:1 mixture of Hypnorm® (fluanisonum 10 mg $ml^{-1}$ and fentanylum 0.2 mg $ml^{-1}$) and Dormicum® (midazolam 5 mg $ml^{-1}$) 1.8 $ml^{-1}$ $kg^{-1}$ administered as a single i.p. injection. With the aid of a 22G microsyringe (Unimetrics, Shorewood, Illinois, USA) fitted to the micromanipulator of a stereotactic frame $2 \times 10^4$ cells were transplanted stereotactically to the right caudate nucleus of the rat brain. The animals were housed in a controlled environment (12 h light/12 h dark) with pellets and water freely available. The experiments were approved by the local ethics committee with the recommendation of using a limited number of animals. Eight rats were chosen at random for IL-2 treatment, 10 rats for histamine treatment, eight rats for treatment with a combination of IL-2 and histamine and 20 rats served as untreated controls.

### IL-2 and histamine administration

IL-2 was continuously delivered by a subcutaneous infusion using Alzet 2002 micro-osmotic pumps (Alza Corporation, Palo Alto, CA, USA). The pumps containing 200 μL IL-2 at a concentration of 1 800 000 IU $ml^{-1}$ were implanted subcutaneously on day 6 after tumour implantation, when small solid tumours are known to have been formed (Bergenheim et al, 1994). The micro-osmotic pump system makes it possible to deliver 0.5 μl $h^{-1}$ for 14 days (Henriksson et al, 1992). The daily dose delivered in this way was 21 600 IU 24 $h^{-1}$, or approximately 66 000 IU $kg^{-1}$ 24 $h^{-1}$. The IL-2 infusion was continued until day 20 after tumour implantation. Histamine was administrated as daily subcutaneous injections at a dose of 4 mg $kg^{-1}$. Histamine treatment started the day after implantation of IL-2 pumps and continued until the termination of the experiment.

### Tumour growth

The study was terminated on day 24 after tumour implantation, when animals were decapitated. The brains were immediately carefully dissected and fixed in formaline. Tumours were measured using a microcalliper. Tumour volume was calculated using the formula for the ellipsoid ($r1 \times r2 \times r3 \times 4/3$) where the radius in the sagittal plane was approximated to be the same as the coronal radius. Brains were thereafter fixed cold in 70% ethanol until paraffin embedded. For routine histological staining and immunohistochemistry 4 μm sections were cut using a sledge microtome. For immunohistochemistry, tissue sections were mounted on polylysin coated slides.

### Vascular staining and assessment of microvascular density

Vessels in the BT4C brain tumours were immunohistochemically stained for factor VIII and quantified manually using a method originally presented by Weidner and coworkers (Weidner, 1993).

Sections chosen for immunohistochemical staining were deparaffinized, rehydrated and permeabilized in 0.05% protease P27 at 37°C for 30 min. After blocking with normal goat sera sections were incubated for 1 h at room temperature with a polyclonal rabbit anti-human factor VIII antibody (DAKO A/S, Denmark) diluted 1:200. After washing in PBS, sections were incubated with biotinylated goat anti-rabbit antibody and avidin linked alkaline phosphatase (Vector ABC-AP, Vector, Burlingame, CA, USA). Sections were developed using alkaline phosphatase reagent (Vector) and finally mounted using gelatin-glycerol.

Assessment of microvascular density (MVD) was performed by manual counting in selected areas with high vascular density (hot-spots). Each tumour was scanned at low magnification and four hot-spots were chosen for MVD quantification. All stained objects within a 200× field (1.23 $mm^2$) were counted using a standard light microscope (Axiophot, Zeiss, Germany). Each hot-spot was counted twice and the arithmetical mean in each spot was used to calculate the mean MVD for each tumour section, which was used for further statistical analysis. MVD was expressed as number of vessels $mm^{-2}$.

In order to get quantitative information on tumour vessel size, the sections were also analysed using a computerized image analysis system (CIAS). The system used consists of a standard microscope (Axiophot, Zeiss, Oberkochen, Germany) fitted with a high resolution digital camera (ProgRes 3000, Kontron Elektronik GmbH, Eching bei München, Germany) under control of WinCam 1.4 software (CCD-Videometrie, Unterscheissheim, Germany) in a PC computer. Images were thereafter analysed using KS-400 2.00 software (Kontron Elektronik). The image analysis procedure was automatized by a KS-400 macro with individual threshold setting for each section. Threshold setting was subjectively verified by superimposing the contours of the obtained binary image on the original. If detectable mismatch was found, the procedure of thresholding was redone until congruence was achieved. Four vascular hot-spots was data from each spot at 200× magnification (area 0.307 $mm^2$). For each vessel in each area the perimeter, area, maximal diameter and minimal diameter was measured and stored in a database. Means for each tumour were calculated and used for further statistical analysis. Frequency distributions of the vessel areas were based on the entire database divided into the different groups of treatment.

### VEGF expression

The mRNA expression of VEGF was evaluated by in situ hybridization in histological sections from at least four different tumours in each treatment group. In situ hybridization for vascular endothelial growth factor (VEGF) was performed mainly as previously described (Lindgren et al, 1997), modified for formalin fixed paraffin embedded sections. Briefly, specific polymerase chain reaction (PCR) primers were selected for rat glioma derived VEGF and reverse transcriptase (RT) PCR was performed on 1 μg tumour mRNA. Following cloning into a commercially available plasmid (Bluescript SK +/–, Stratagene, La Jolla, CA, USA) a digoxin (DIG)-labelled RNA probe was obtained by using a DIG-RNA kit (Boehringer Mannheim, Stockholm, Sweden) according to the manufacturer's recommendations. Formalin fixed sections were dewaxed in xylene and rehydrated through a graded ethanol series. Thereafter, sections were fixated in 4% paraformaldehyde (PFA)/phosphate buffered saline (PBS), incubated in 0.2 M HCl, acetylated and treated with proteinase K 25 μg $ml^{-1}$ to further break formalin-induced protein changes. In situ hybridization was performed with DIG-labelled RNA probes at a concentration of 50 ng 100 $\mu l^{-1}$ at 70°C overnight. The DIG-labelled probes were thereafter tracked by incubating sections with anti-DIG fabs (Boehringer Mannheim). Development was carried out at 30°C with 5-Bromo-4-Chloro-3-Inolyl-Phosphate (Boehringer Mannheim), 50 mg $ml^{-1}$ 4-Nitro Blue Tetra-zoliumchloride (Boehringer Mannheim) for 12 h. Finally, sections were mounted in gelatine glycerol medium.

### Apoptosis

In situ end labelling (ISEL) is a method for visualization of apoptotic cells in terms of DNA strand-breaks. The protocol of Wijsman and coworkers was used (Wijsman et al, 1993). Sections

© 2000 Cancer Research Campaign

3–4 µm thick were cut from the paraffin-embedded specimens according to routine histology procedures. After deparaffinization and rehydration, the sections were heated twice in SSC (sodium chloride 17.5 g, sodium citrate 8.8 g $l^{-1}$ water, pH 7.0; Merck Darmstadt, Germany) at 80°C for 20 min and subsequently washed thoroughly in distilled water. To enable enzymatic incorporation of nucleotides, the sections were digested in 0.5% pepsin in hydrochloric acid (pH 2) for 15 min with gentle shaking in a 37°C water bath. The digestion was stopped by washing several times in tap water and then washed in buffer for 5 min. After drying, the sections were incubated for 1 h at 15°C with buffer containing 0.01 mM dATP, dGTP, dCTP and 0.01 mM biotin dUTP (Boehringer Mannheim) along with 4 U $ml^{-1}$ DNA polymerase 1 (Sigma, USA). Endogenous peroxidase was blocked for 5 min in PBS with 0.1% hydrogen peroxide, and the sections were then washed twice in PBS (0.1% hydrogen peroxide). The sections were incubated with avidin dissolved in PBS with 1% BSA (bovine serum albumin) and 0.5% Tween 20 (Boehringer Mannheim) for 30 min at room temperature before developing with diaminobenzidine. In negative controls, DNA polymerase was excluded from the nucleotide polymerase mix. Normal rat prostate, 3 days after castration, was used as a positive control. The number of ISEL-positive cells was quantified in the light microscope. Apoptotic index was expressed as the fraction apoptotic cells of the total number of cells, expressed as percent.

### Tumour infiltrating macrophages

Sections chosen for immunohistochemical staining of macrophages were deparaffinized in xylene, rehydrated in graded ethanol series and washed in deionized water. The tissue was thereafter permeabilized by microwave heating in 0.01 M citrate buffer pH 6.0 for 5 min × 4 followed by washing in PBS for 5 min × 4. Endogenous peroxidase activity was inhibited by incubation in 3% $H_2O_2$ methanol for 20 min. Slides were then incubated with normal goat serum for 20 min prior to overnight incubation at 8°C with mouse anti-rat ED-1 antibody (MCA 341, Serotec Ltd, Oxford, UK) diluted 1:200. After washing in PBS 5 min × 4 slides were incubated with a biotinylated goat anti-mouse antibody (Vector Laboratories) for 30 min and washed in PBS 5 min × 4 again. Sections were subsequently incubated with avidin linked peroxidase (Vector ABC) for 20 min according to the vendor's recommendations. Development was finally performed using 3,3′-diaminobenzidine (DAB) tablets (Sigma) according to the manufacturer's recommendations. After 5 min wash in tap water, slides were mounted in gelatine glycerol. Immunohistochemistry for ED-1 was performed on all tumours and staining was semiquantitatively assessed by two observers as missing (0), weak (1), moderate (2) or pronounced (3).

### Tumour blood flow

To analyse blood flow in tumour tissue and normal brain, a model utilizing single-fibre laser Doppler flowmetry was set up. Recordings of tumour as well as normal cortex perfusion were done simultaneously with a dual-channel Laser Doppler flowmeter (Periflux PF4000, Perimed, Järfälla, Sweden) prior to and after administration of histamine. The laser Doppler signal was transferred on line to a personal computer equipped with the Perisoft software package (Perimed).

The left femoral artery was catheterized and the catheter was connected to a pressure transducer for online registration of systemic blood pressure. Immediately thereafter, animals were fixed in a stereotactic frame (Kopf 900, David Kopf, Tujunga, CA, USA) and the skull bone was exposed with a sagittal incision. Care was taken to obtain optimal haemostasis. A burr hole 3.5 mm to the left of bregma was made contralateral to the tumour implantation burr hole in the right hemisphere bone to expose the normal left hemisphere. The dura mater was incised carefully. Two single-fibre laser Doppler probes (Probe 418, Perimed) fitted to the micromanipulator arms of the stereotactic instrument were thereafter advanced to a depth of 2 mm in the tumour and contralateral brain hemisphere, respectively.

When adequate signal from the laser Doppler flowmeter was obtained, the preparation was left undisturbed for 10 min to allow the system to equilibrate. A baseline flow recording of at least 5 min was performed prior to administration of histamine 4 mg $kg^{-1}$ s.c. Observation of changes in blood flow was continued for at least 10 min after histamine administration. The maximal perfusion change post-histamine administration was thereafter compared to the mean baseline perfusion value using the Perisoft software package (Perimed). Vascular resistance was calculated by dividing mean arterial pressure with obtained flow values. Four animals were randomly chosen for blood flow measurements 20 days after tumour implantation.

### Statistical analysis

Values are expressed as means ± SE unless otherwise indicated. For comparisons between groups Mann–Whitney U test was used. For comparison of manual and computerized vascular counts, linear regression was used. Statistical analysis was performed using the StatView 4.5 software (Abacus Concepts, Berkely, CA, USA) for the Macintosh (Apple Computer, Cupertino, CA, USA) computer.

### RESULTS

### Tumour growth

The combination of IL-2 and histamine reduced tumour volume (95 ± 11 $mm^3$) significantly when compared to untreated controls

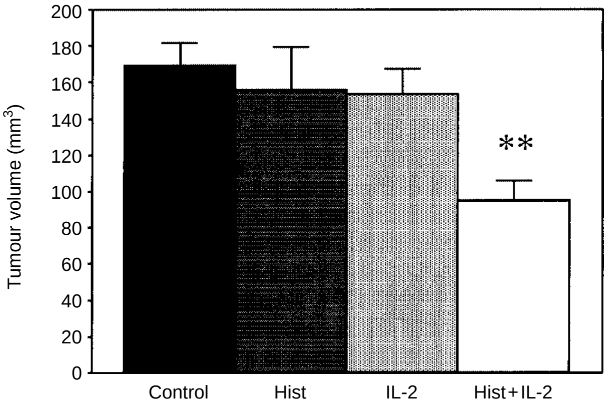

**Figure 1** Brain tumour volume ($mm^3$) in control animals and animals treated with histamine (Hist), IL-2 alone (IL-2) or histamine combined with IL-2 (Hist + IL-2). Values are given as means ± SE. ***P* < 0.01 compared to control

© 2000 Cancer Research Campaign

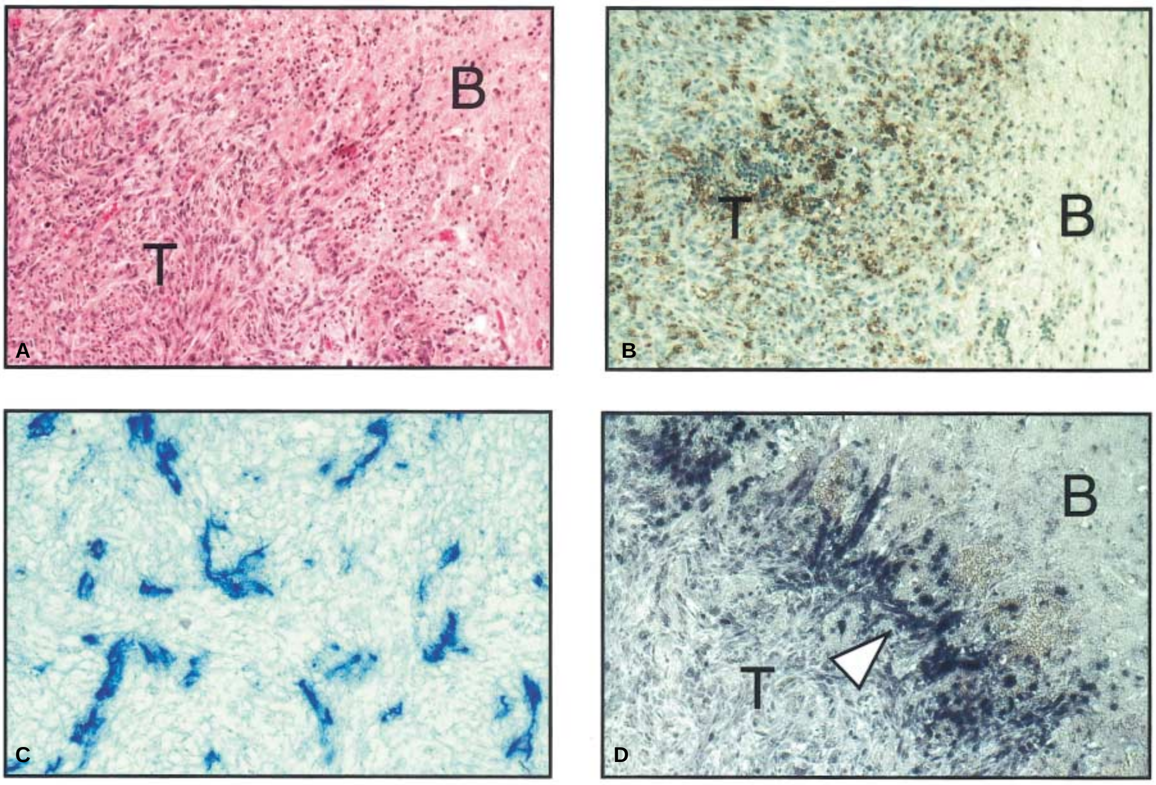

**Figure 2** Photomicrographs showing (**A**) haematoxylin-eosin stain of a control tumour (×25), (**B**) ED-1 macrophage immunohistochemical stain (×25), (**C**) factor VIII immunohistochemistry of a vascular hot-spot in a control animal (×200) and (**D**) VEGF in situ hybridization demonstrating the high expression in tumour border (arrow) in a control animal (×25). T indicates tumour and B normal brain tissue

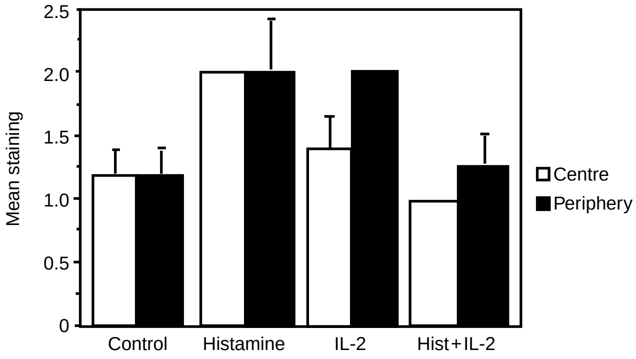

**Figure 3** Semi-quantitative assessment of macrophages detected by ED-1 immunostaining in the centre and periphery of tumours. Staining classified as missing (0), low (1), moderate (2) or high (3). Values are given as means ± SE

(169 ± 12 mm$^3$) ($P$ < 0.01). Histamine or IL-2 alone did not affect tumour growth (156 ± 3 mm$^3$ and 153 ± 14 mm$^3$, respectively). (Figure 1).

### Morphology

Manifest tumours were identified in all animals (Figure 2A). ED-1 positive macrophages were identified in all examined tumours (Figure 2B). ED-1-positive cells were mainly found infiltrating the invasive border of the tumour where haemorrhages were common. Histamine and/or IL-2 treated animals had a strong tendency of an increase in ED-1-positive cells, especially in the tumour periphery (Figure 2B, Figure 3) No central necrotic areas were found and factor VIII-positive microvessels were found throughout the tumour (Figure 2C). Vascular density was most intensive in the periphery of tumours, where microvascular hot-spots most commonly were identified. In situ hybridization of VEGF showed no obvious changes following any of the treatments (Figure 2D). The apoptotic index in the control group was 5.3 ± 3% and no significant difference between the groups was observed. In the positive control, i.e. rat prostate, a significant number of the epithelial cells displayed characteristic features of apoptotic death 3 days after castration (not shown).

### Microvascular density

Intratumoural microvascular density was significantly decreased by the combination of IL-2 and histamine when compared to untreated controls when counted manually (Table 1). Mean MVD in the control group was 205 ± 13 vessels mm$^{-2}$ compared to the IL-2/histamine group where the average MVD was 152 ± 4.5 vessels mm$^{-2}$ ($P$ < 0.01). Neither histamine nor IL-2 did affect tumour MVD significantly when given alone and the groups had a mean MVD of 165 ± 16 vessels mm$^{-2}$ and 174 ± 10 vessels mm$^{-2}$ respectively.

MVD assessed by CIAS showed good correlation to the manually obtained data ($r$ = 0.67) and the combination of IL-2 and histamine was found to decrease MVD also when analysed with the

*© 2000 Cancer Research Campaign* 

**Table 1** Computerized image analysis of microvascular density (MVD), perimeter, area, minimum diameter and maximum diameter in treated and not treated intracerebral rat glioma

| Group | IA MVD (v mm$^{-2}$) | Perimeter (μm) | Area (μm$^2$) | D Min (μm) | D Max (μm) |
|---|---|---|---|---|---|
| Controls | 200 ± 18 | 108 ± 7.0 | 217 ± 24 | 13 ± 0.7 | 26 ± 1.4 |
| Histamine | 175 ± 14 | 78 ± 5.9* | 110 ± 13* | 9.8 ± 0.5* | 20 ± 1.1** |
| IL-2 | 154 ± 5.5 | 98 ± 4.3 | 180 ± 16 | 12 ± 0.5 | 25 ± 1.0 |
| Hist + IL-2 | 128 ± 5.7** | 135 ± 4.6* | 336 ± 18** | 15 ± 0.5* | 32 ± 1.0** |

Values are given as means ± SE. *$P$ < 0.05, **$P$ < 0.01 compared to control

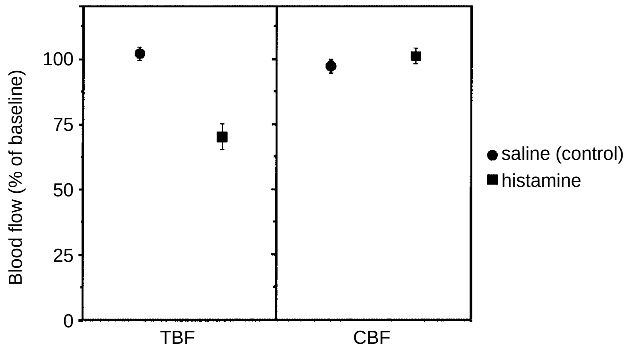

**Figure 4** Tumour blood flow (TBF) and cerebral blood flow (CBF) after administration of saline (control) and histamine as assessed by single-fibre laser Doppler flowmetry (LDF). Values are given as mean percent of baseline ± SE

CIAS. Noteworthy, individual tumour vessel size decreased significantly when histamine was administered. Mean vessel area, perimeter and diameter all decreased significantly in the histamine group. The combination of IL-2 and histamine was found to increase all vessel size parameters concomitantly with the decrease in MVD (Table 1).

### Tumour blood flow

Histamine administration rapidly decreased mean arterial pressure from 89 ± 6 to 58 ± 4 mmHg followed by a significant decrease in tumour perfusion (Figure 4). The tumour vascular resistance was not altered following histamine administration while cerebral vascular resistance decreased. Vascular resistance following histamine injection expressed as percent of baseline was 97 ± 6% in tumour and 65 ± 2% in the brain. Systemic blood pressure and tumour perfusion returned to pre-histamine values after approximately 15 min. No change in normal brain perfusion was seen during the experiment.

### DISCUSSION

This study demonstrated for the first time, as far as we know, that histamine in combination with IL-2 reduced the growth of an intracerebral malignant glioma. The results are in accordance with earlier observations that manipulation of the immune system with histamine and IL-2 inhibits growth of extracranial experimental tumours (Johansson et al, 1998). In addition to earlier suggested immunological effects (Hellstrand and Hermodsson, 1990; Hellstrand et al, 1994a), the present results propose that interaction with tumour angiogenesis and tumour perfusion may be mechanisms involved in the action of the combination of IL-2 and histamine. Noteworthy, treatment with either IL-2 or histamine alone was without any detectable effects on tumour growth.

Despite extensive efforts, a huge number of various pharmacotherapeutical approaches have shown a lack of meaningful clinical benefits in malignant glioma. Immunotherapy has given some interesting but so far clinically questionable results (Danaila et al, 1993; Hayes et al, 1995; Kruse et al, 1997). IL-2 alone, or used for adoptive immunotherapy in conjunction with lymphokine-activated killer (LAK) or tissue infiltrating lymphocytes (TIL), showed some interesting experimental results (Tzeng et al, 1990; Saris et al, 1992) but have demonstrated controversial results in the clinical setting (Barba et al, 1989*b*; Lillehei et al, 1991). The disappointing effects of immunotherapy in the management of malignant glioma may be related to the feature of the intracerebral compartment as immunologically privileged. Poor target recognition, limited major histocompatibility antigen expression, incomplete trafficking of immuno-effector cells across the blood–brain barrier and redeeming actions of tumour cells that hamper cytolytic activity of effector cells, might all contribute to difficulties in brain tumour immunotherapy (Fontana et al, 1984; Tzeng et al, 1990; Saris et al, 1992). The previously discussed immunologic mechanisms of action of histamine (Hellstrand et al, 1990; 1994*a*) could thus be of interest to explain the present obtained results.

In this study, treatment with histamine and IL-2 in combination also significantly reduced the density of tumour microvessels. Both IL-2 and histamine are known to affect tumour vasculature by increasing permeability (Alexander et al, 1989; Nomura et al, 1994) but the finding that histamine and IL-2 in combination decreases tumour MVD is novel, according to our knowledge. Recent investigations have also suggested that the tumour vasculature may be an important target for activated NK cells, and VEGF has been shown to increase adhesion of NK cells to tumour endothelium (Melder et al, 1996). Therefore, it may be proposed that inhibition of tumour angiogenesis or damage to manifest tumour vessels may be an additional mechanism of action for histamine in combination with IL-2. Individual tumour vessel size was found to increase concomitantly with the observed decrease in tumour MVD after combination treatment with histamine and IL-2. This may reflect the fact that fewer microvessels are generated in the combination-treated tumours and one may speculate that the combination of histamine and IL-2 is angiostatic rather than angiotoxic. The lack of patchy haemorrhagic necrosis in treated tumours supports this speculation. Anyhow, the molecular mechanisms behind the decrease in MVD remain unclear and further studies are needed. In this study we were not able to show any obvious change in the pattern of VEGF expression or apoptotic cell death. Thus, it might indicate that this treatment does not

 © 2000 Cancer Research Campaign interfere with VEGF-mediated angiogenesis. However, VEGF is shown to increase endothelial cell surface expression of adhesion molecules and thus may facilitate NK cells to bind and kill endothelial cells (Melder et al, 1996). In our tumour model VEGF expression is increased at the tumour border and this may be a prerequisite for IL-2-activated NK cells to attach to tumour endothelium (Lindgren et al, 1997).

The failure of IL-2 alone to induce a detectable tumour response in our study finds support in recent published studies (Fathallah Shaykh et al, 1998) and further emphasizes the complexity of immunotherapy in malignant glioma. An IL-2 dose of the same magnitude as used in this study has previously been seen to cause significant anti-tumour effects in extracranially located rat prostatic carcinoma and thus most probably demonstrates that an effective systemic concentration of IL-2 was achieved (Henriksson et al, 1992; Johansson et al, 1998). However, even if the dose of IL-2 was relatively high, one explanation of the lack of effect of IL-2 in our orthotopic glioma model could be that the concentration of IL-2 in the intracerebral compartment was not optimal. Previously, it has been shown that histamine has the ability to increase vascular permeability in an experimental intracerebral glioma, but not in normal brain vessels (Nomura et al, 1994). The addition of histamine could thus increase the possibility to achieve a sufficient concentration of IL-2 in the brain tumour and also to enhance the permeability of blood–brain-tumour barrier for immunocompetent cells. Subsequently, an effective immunological reaction could be achieved in the intracranial tumour compartment. In accordance with these assumptions it could be noticed that intratumoural injection of IL-2 in human glioma grade IV in nude mice caused growth retardation (Weber et al, 1989).

All examined tumours in this study were positive for the specific marker for a cell-surface antigen on macrophages – phagocytes, ED-1 (Dijkstra et al, 1985). The ED-1 positive cells were mainly found in the invasive border of the tumour where the microvascular density and VEGF expression is known to be high (Lindgren et al, 1997). It is therefore tempting to speculate that histamine reduces the inhibitory effects of phagocytes on IL-2-stimulated NK cells, which may have the ability to block NK cells infiltrating the tumour, as previously discussed (Hellstrand et al, 1990; 1994*a*). In this respect it is of interest to recall the observation that cytotoxic lymphocyte responses in rat glioma have consistently been shown against NK cell targets (Holladay et al, 1992). Since tumour-associated macrophages are known to either stimulate angiogenesis (via tumour necrosis factor α) or inhibit angiogenesis (via GM-CSF and plasminogen activator inhibitor-2) it can be speculated that histamine might promote the anti-angiogenic effects (Joseph and Isaacs, 1998) as seen in this study. The release of secondary cytokines following IL-2 may further contribute to the observed complex effects seen. It is also of interest that the high VEGF expression observed in the invasive tumour border correlates with the presence of ED-1 positive phagocytes. The presence of phagocytes in the tumour region where NK cell-mediated endothelial cell killing is supposed to be highest may explain the lack of effect on tumour vasculature of IL-2 alone.

Histamine also induces a rapid and temporary decline in tumour blood flow (TBF) without affecting normal brain perfusion. The decrease in TBF is clearly an effect of reduced perfusion pressure due to transient systemic hypotension. Tumour vascular resistance remains unchanged while resistance in normal brain tissue decreases after histamine administration. This finding illustrates the lack of autoregulation in tumour tissue and the possibility to modulate tumour blood flow with alterations in systemic blood pressure. A reduction of tumour blood flow of the magnitude seen in this experiment could possibly induce vascular shut down and hypoxia, which might explain the haemorrhagic necrosis observed by other authors following histamine treatment (Burtin et al, 1982; Johansson et al, 1998). However, in this study we did not observe any morphological changes indicating acute vascular damage inflicted by the treatment. The lack of haemorrhagic necrosis in our study may be due to the relatively short duration of histamine treatment allowed in the fast growing intracerebral tumour model, compared to the more slowly growing subcutaneous tumour model used (Johansson et al, 1998).

Histamine and IL-2 are both commercially available for human use and the clinical usefulness of this treatment combination is to be evaluated. However, in the clinical setting there are some obvious concerns. First, corticosteroids are known to down-regulate VEGF expression and the use of steroids may therefore also decrease the NK cell-mediated endothelial cell killing (Bruce et al, 1987; Heiss et al, 1996). Second, the decrease in tumour blood flow seen after histamine administration should be considered if histamine is used in combination with other agents aimed at tumour cells. Third, the potential risk of cerebral oedema and increased intracranial pressure due to increased blood vessel permeability, induced both by histamine and IL-2, must also be considered in the clinical situation (Barba et al, 1989*b*).

In conclusion, these results suggest that the novel combination of IL-2 and histamine could be of interest in improving the therapeutic outcome in malignant glioma. In addition to direct immunological mechanisms it is suggested that inhibition of tumour angiogenesis may be a new mechanism of action for the combination of IL-2 and histamine. However, further studies are needed to delineate the mechanisms of action and to find the optimal schedule of this treatment before the value of this approach can be established.

### ACKNOWLEDGEMENTS

The study was supported by grants from the Swedish Society against Cancer, the Lion's Cancer Research Foundation, Umeå, Sweden and Umeå University.

### REFERENCES

Alexander JT, Saris SC and Oldfield EH (1989) The effect of interleukin-2 on the blood–brain barrier in the 9L gliosarcoma rat model. *J Neurosurg* **70**: 92–96

Asea A, Hermodsson S and Hellstrand K (1996) Histaminergic regulation of natural killer cell-mediated clearance of tumour cells in mice. *Scand J Immunol* **43**: 9–15

Barba D, Saris SC, Holder C, Rosenberg SA and Oldfield EH (1989*a*) Intratumoral LAK cell and interleukin-2 therapy of human gliomas. *J Neurosurg* **70**: 175–182

Barba D, Saris SC, Holder C, Rosenberg SA and Oldfield EH (1989*b*) Intratumoral LAK cell and interleukin-2 therapy of human gliomas. *J Neurosurg* **70**: 175–182

Bergenheim AT, Elfversson J, Gunnarsson P-O, Edman K, Hartman M and Henriksson R (1994) Cytotoxic effect and uptake of estramustine in a rat glioma model. *Int J Oncol* **5**: 293–299

Bruce JN, Criscuolo GR, Merrill MJ, Moquin RR, Blacklock JB and Oldfield EH (1987) Vascular permeability induced by protein product of malignant brain tumors: inhibition by dexamethasone. *J Neurosurg* **67**: 880–884

© 2000 Cancer Research Campaign

Burtin C, Scheinmann P, Salomon JC, Lespinats G and Canu P (1982) Decrease in tumour growth by injections of histamine or serotonin in fibrosarcoma-bearing mice: influence of H1 and H2 histamine receptors. *Br J Cancer* **45**: 54–60

Caligiuri MA, Murray C, Robertson MJ, Wang E, Cochran K, Cameron C, Schow P, Ross ME, Klumpp TR, Soiffer RJ, Smith KA and Ritz J (1993) Selective modulation of human natural killer cells in vivo after prolonged infusion of low dose recombinant interleukin 2. *J Clin Invest* **91**: 123–132

Danaila L, Ghyka G and Ursaciuc C (1993) Interleukin-2 (IL-2) in the treatment of malignant brain tumors (glioblastomas). *Rom J Neurol Psychiatry* **31**: 195–206

Dijkstra CD, Dopp EA, Joling P and Kraal G (1985) The heterogeneity of mononuclear phagocytes in lymphoid organs: distinct macrophage subpopulations in the rat recognized by monoclonal antibodies ED1, ED2 and ED3. *Immunology* **54**: 589–599

Fathallah Shaykh HM, Gao W, Cho M and Herrera MA (1998) Priming in the brain, an immunologically privileged organ, elicits anti-tumor immunity. *Int J Cancer* **75**: 266–276

Fleshner M, Watkins LR, Redd JM, Kruse CA and Bellgrau D (1992) A 9L gliosarcoma transplantation model for studying adoptive immunotherapy into the brains of conscious rats. *Cell Transplant* **1**: 307–312

Fontana A, Hengartner H, de Tribolet N and Weber E (1984) Glioblastoma cells release interleukin 1 and factors inhibiting interleukin 2-mediated effects. *J Immunol* **132**: 1837–1844

Glick RP, Lichtor T, Mogharbel A, Taylor CA and Cohen EP (1997) Intracerebral versus subcutaneous immunization with allogeneic fibroblasts genetically engineered to secrete interleukin-2 in the treatment of central nervous system glioma and melanoma. *Neurosurgery* **41**: 898–906

Hayes RL, Koslow M, Hiesiger EM, Hymes KB, Hochster HS, Moore EJ, Pierz DM, Chen DK, Budzilovich GN and Ransohoff J (1995) Improved long term survival after intracavitary interleukin-2 and lymphokine-activated killer cells for adults with recurrent malignant glioma. *Cancer* **76**: 840–852

Heiss JD, Papavassiliou E, Merrill MJ, Nieman L, Knightly JJ, Walbridge S, Edwards NA and Oldfield EH (1996) Mechanism of dexamethasone suppression of brain tumor-associated vascular permeability in rats. Involvement of the glucocorticoid receptor and vascular permeability factor. *J Clin Invest* **98**: 1400–1408

Hellstrand K and Hermodsson S (1990) Synergistic activation of human natural killer cell cytotoxicity by histamine and interleukin-2. *Int Arch Allergy Appl Immunol* **92**: 379–389

Hellstrand K, Asea A and Hermodsson S (1990) Role of histamine in natural killer cell-mediated resistance against tumor cells. *J Immunol* **145**: 4365–70

Hellstrand K, Asea A, Dahlgren C and Hermodsson S (1994*a*) Histaminergic regulation of NK cells. Role of monocyte-derived reactive oxygen metabolites. *J Immunol* **153**: 4940–4947

Hellstrand K, Naredi P, Lindner P, Lundholm K, Rudenstam CM, Hermodsson S, Asztely M and Hafstrom L (1994*b*) Histamine in immunotherapy of advanced melanoma: a pilot study. *Cancer Immunol Immunother* **39**: 416–419

Henriksson R, Widmark A, Bergh A and Damber JE (1992) Interleukin-2-induced growth inhibition of prostatic adenocarcinoma (Dunning R3327) in rats. *Urol Res* **20**: 189–191

Henriksson R, Nilsson S, Colleen S, Wersall P, Helsing M, Zimmerman R and Engman K (1998) Survival in renal cell carcinoma – a randomized evaluation of tamoxifen vs interleukin 2, alpha-interferon (leucocyte) and tamoxifen. *Br J Cancer* **77**: 1311–1317

Holladay FP, Lopez G, De M, Morantz RA and Wood GW (1992) Generation of cytotoxic immune responses against a rat glioma by in vivo priming and secondary in vitro stimulation with tumor cells. *Neurosurgery* **30**: 499–504

Johansson M, Bergenheim AT, Henriksson R, Koskinen LO, Vallbo C and Widmark A (1997) Tumor blood flow and the cytotoxic effects of estramustine and its constituents in a rat glioma model. *Neurosurgery* **41**: 237–243

Johansson S, Landstrom M, Hellstrand K and Henriksson R (1998) The response of Dunning R3327 prostatic adenocarcinoma to IL-2, histamine and radiation. *Br J Cancer* **77**: 1213–1219

Joseph IB and Isaacs JT (1998) Macrophage role in the anti-prostate cancer response to one class of antiangiogenic agents [see comments]. *J Natl Cancer Inst* **90**: 1648–1653

Kruse CA, Cepeda L, Owens B, Johnson SD, Stears J and Lillehei KO (1997) Treatment of recurrent glioma with intracavitary alloreactive cytotoxic T lymphocytes and interleukin-2. *Cancer Immunol Immunother* **45**: 77–87

Lillehei KO, Mitchell DH, Johnson SD, McCleary EL and Kruse CA (1991) Long-term follow-up of patients with recurrent malignant gliomas treated with adjuvant adoptive immunotherapy. *Neurosurgery* **28**: 16–23

Lindgren M, Johansson M, Sandstrom J, Jonsson Y, Bergenheim AT and Henriksson R (1997) VEGF and tPA co-expressed in malignant glioma. *Acta Oncol* **36**: 615–618

Ljungberg B and Henriksson R (1997) Immunotherapy of metastatic renal cell carcinoma. *Curr Opin Urol* **7**: 252–258

Melder RJ, Koenig GC, Witwer BP, Safabakhsh N, Munn LL and Jain RK (1996) During angiogenesis, vascular endothelial growth factor and basic fibroblast growth factor regulate natural killer cell adhesion to tumor endothelium [see comments]. *Nat Med* **2**: 992–997

Nomura T, Ikezaki K, Matsukado K and Fukui M (1994) Effect of histamine on the blood-tumor barrier in transplanted rat brain tumors. *Acta Neurochir (Wien)* **60**: 400–402

Saris SC, Spiess P, Lieberman DM, Lin S, Walbridge S and Oldfield EH (1992) Treatment of murine primary brain tumors with systemic interleukin-2 and tumor-infiltrating lymphocytes. *J Neurosurg* **76**: 513–519

Takai N, Tanaka R, Yoshida S, Hara N and Saito T (1988) In vivo and in vitro effect of adoptive immunotherapy of experimental murine brain tumors using lymphokine-activated killer cells. *Cancer Res* **48**: 2047–2052

Tzeng JJ, Barth RF, Clendenon NR and Gordon WA (1990) Adoptive immunotherapy of a rat glioma using lymphokine-activated killer cells and interleukin 2. *Cancer Res* **50**: 4338–4343

Weber F, List J, Rommel T, Menzel J, Symas J, Pohl U, Mohr H, Schmitz R and Amue B (1989) Influence of interleukin II on xenotransplanted grade 3 to 4 glioma in nude mice. *Strahlenther Onkol* **165**: 556–558

Weidner N. (1993) Tumor angiogenesis: review of current applications in tumour prognostication. *Semin Diagn Pathol* **10**: 302–313

Wijsman JH, RJonker R, Keijzer R, DeVelde DJH, Cornel-Issue CJ & Dierendonck JHV (1993) A new method to detect apoptosis in paraffin sections: In situ end-labelling of fragmented DNA. *J Histochem Cytochem* **41**: 7–12

 *© 2000 Cancer Research Campaign*
